# Supplementary figures and images for: A single dose of antibody-drug conjugate cures a stage 1 model of African trypanosomiasis
Source: PLoS Negl Trop Dis. 2019 May 23;13(5):e0007373. doi: 10.1371/journal.pntd.0007373 (PMC6532856; doi:10.1371/journal.pntd.0007373)

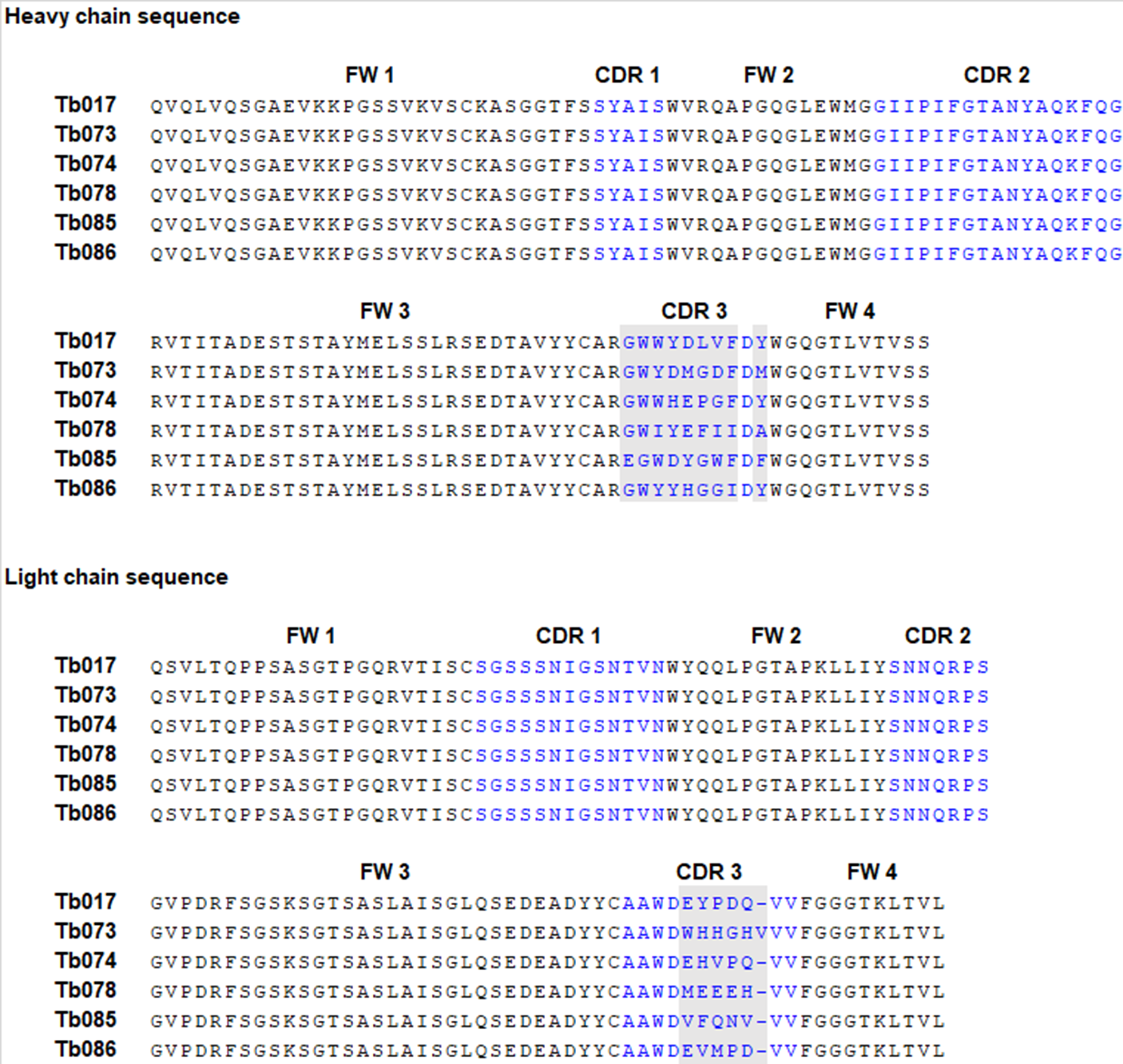

Supplement: S1 Fig — The framework domains (FW) are shown in black and the complementarity-determining regions (CDR1-3) are shown in blue. Sequence variation between scFvs is in CDR3, as annotated by grey boxes. (TIF) [file pntd.0007373.s001.tif]

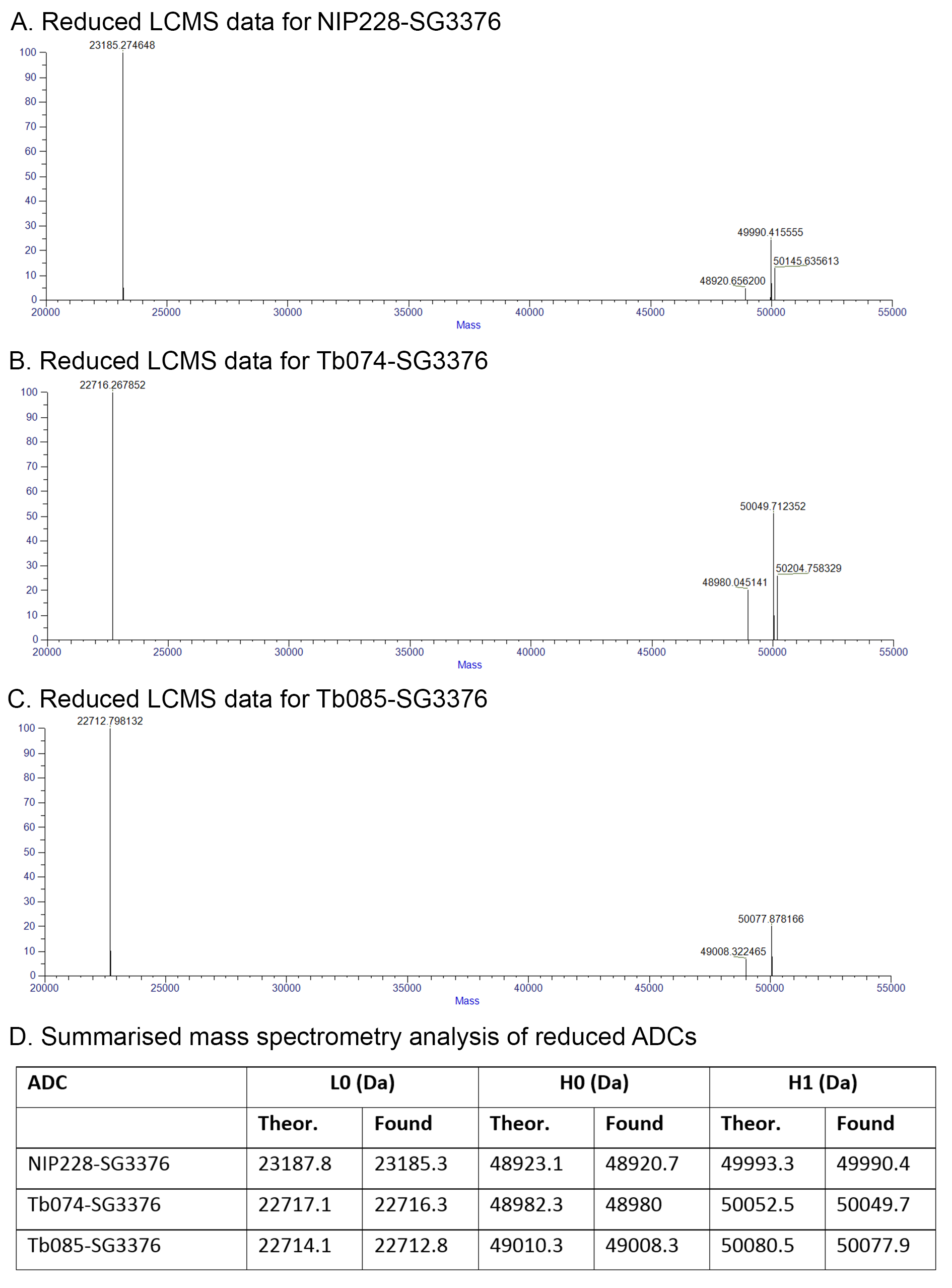

Supplement: S3 Fig — Mass spectrometry analysis of reduced antibody-toxin conjugates was performed using a RSLC UPLC system coupled to an Exactive EMR Orbitrap MS. L0 = unconjugated light chain species, H0 = unconjugated heavy chain species, H1 = conjugated heavy chain species. (TIF) [file pntd.0007373.s003.tif]

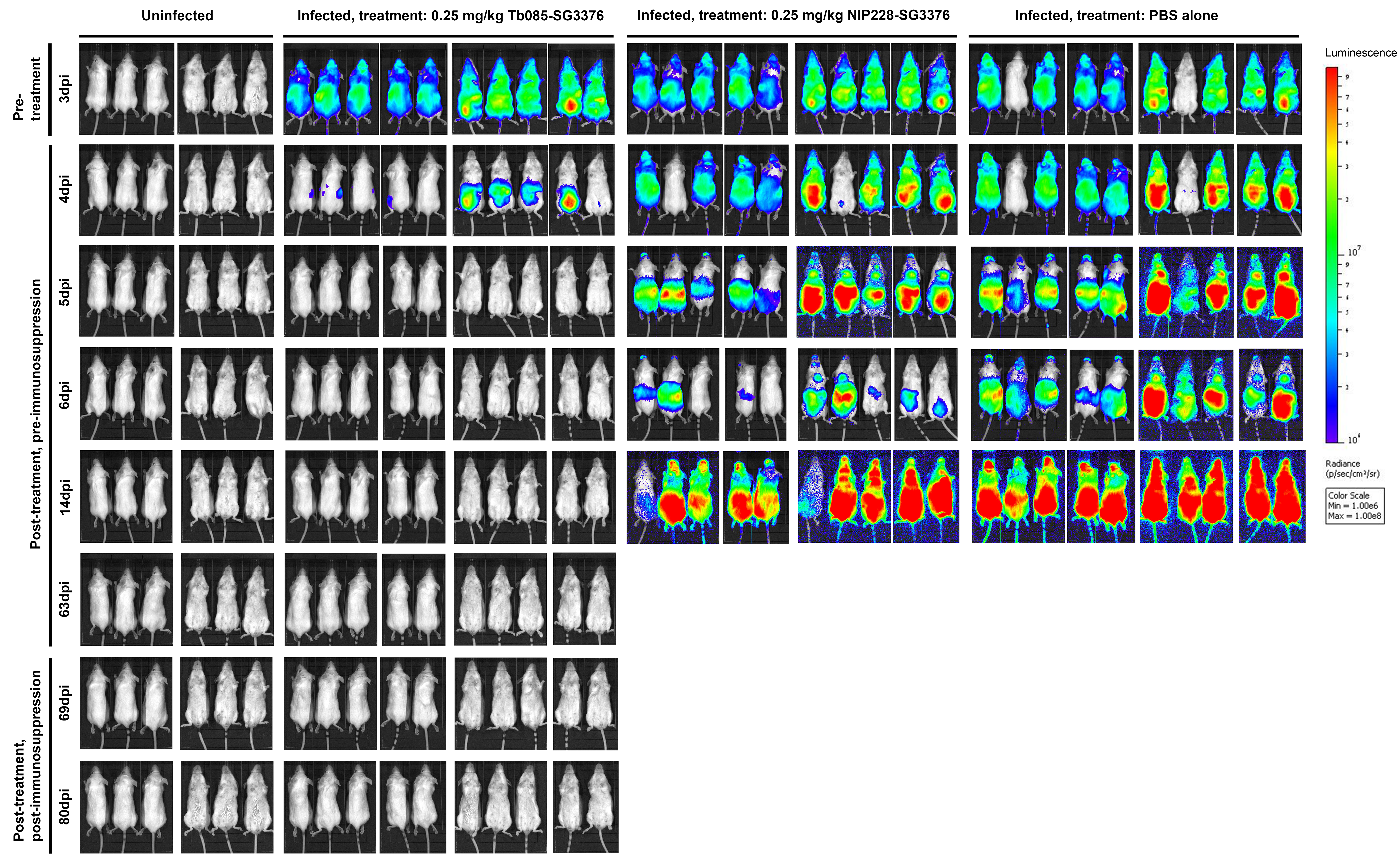

Supplement: S4 Fig — Parasite burden in mice infected with pleomorphic T. b. brucei GVR35-VSL2 cells was assessed by BLI following intraperitoneal injection of d-luciferin. BLI was performed prior to any treatment at 3 days post infection (dpi) and then at regular time points following treatment on 3 dpi with (1) Tb085-SG3376 (n = 5), (2) NIP228-SG3376 (n = 5) or (3) PBS alone (n = 5), with selected time points shown here. Uninfected mice were imaged as controls (n = 3). Treatment with Tb085-SG3376 decreased the luminescent signal to that obtained from uninfected control animals within 2 days and this remained the case for the duration of the infection, including following the immunosuppression of Tb085-SG3376- treated mice at 66 dpi. For each group of mice both the dorsal and ventral images are shown. Scale bar represents the photons emitted at any given point on the image. Exposure times range from 0.5 seconds (for heavily burdened mice) to 5 minutes (for uninfected animals). One mouse in the PBS control group had a lower BLI signal than all other infected mice at 3 dpi (S5 Fig). In the image shown here this mouse appears negative, however, this is due to the low exposure time required for adjacent mice. Quantification of the total luminescence from each mouse was also carried out (Fig 4 and S5 Fig). (TIF) [file pntd.0007373.s004.tif]

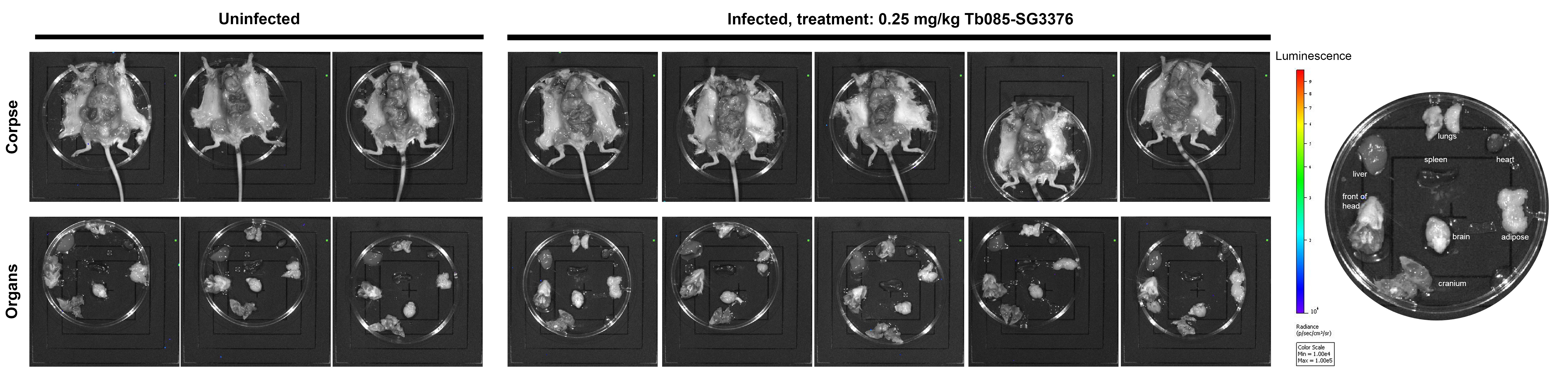

Supplement: S6 Fig — The five mice that were infected with pleomorphic T. b. brucei GVR35-VSL2 cells, treated with 0.25 mg/kg Tb085-SG3376 (3 dpi) and immunosuppressed (66 dpi) were culled at 80 dpi. Post-necropsy, mice corpses and selected organs were assessed by BLI. Consistent with BLI data from live mice, BLI signal was equivalent to the uninfected control mice and so image appears black and white due to the total absence of any luciferase signal. (TIF) [file pntd.0007373.s006.tif]
