# Supplementary material for: A single dose of antibody-drug conjugate cures a stage 1 model of African trypanosomiasis
Source: PLoS Negl Trop Dis. 2019 May 23;13(5):e0007373. doi: 10.1371/journal.pntd.0007373 (PMC6532856; doi:10.1371/journal.pntd.0007373)
Supplement: S1 Table — The IC50 values of toxin SG3199, toxin plus linker SG3249, a control ADC (NIP228-SG3249) and five anti-trypanosome antibody toxin conjugates targeting the T. brucei HpHbR (Tb017-SG3249, Tb073-SG3249, Tb074-SG3249, Tb078-SG3249, Tb085-SG3249) were calculated against T. b brucei wild type (Fig 3). Values in bold are best-fit IC50 values, the range is the 95% confidence intervals. All values are shown to 3 significant figures. (DOCX) [file pntd.0007373.s007.docx]

**S1 Table**

| IC50 (pM) | |
| --- | --- |
|  | ***T. b. brucei*** |
| **SG3199 Toxin** | **0.86** |
|  | (0.69-1.08) |
| **SG3249 Toxin plus Linker** | **236** |
|  | (196-284) |
| **NIP228-SG3249 Control** | **2100** |
|  | (1760-2500) |
| **Tb017-SG3249** | **32.9** |
|  | (26.8-40.3) |
| **Tb073-SG3249** | **85.7** |
|  | (74.6-98.6) |
| **Tb074-SG3249** | **17.3** |
|  | (14.3-21.1) |
| **Tb078-SG3249** | **74.2** |
|  | (64.3-85.7) |
| **Tb085-SG3249** | **9.35** |
|  | (7.59-11.5) |
