# Supplementary material for: A single dose of antibody-drug conjugate cures a stage 1 model of African trypanosomiasis
Source: PLoS Negl Trop Dis. 2019 May 23;13(5):e0007373. doi: 10.1371/journal.pntd.0007373 (PMC6532856; doi:10.1371/journal.pntd.0007373)
Supplement: S2 Table — Monomeric purity was determined by size exclusion chromatography (SEC) and the DAR was determined by RP-HPLC. Both assays were performed on a Shimadzu Nexera UPLC system fitted with a Shimadzu Prominence DAD detector. Data were processed using LabSolutions software. (DOCX) [file pntd.0007373.s008.docx]

**S2 Table**

| **Antibody toxin conjugate** | **SEC** | | | **DAR** |
| --- | --- | --- | --- | --- |
|  | **% HMW** | **% Monomer** | **% LMW** |  |
| **NIP228-SG3376** | 3.2 | 90.1 | 6.7 | 1.77 |
| **Tb074-SG3376** | 5.4 | 92.1 | 2.5 | 1.80 |
| **Tb085-SG3376** | 0 | 100 | 0 | 1.79 |
